# Therapies to Prevent Progression of COVID-19, Including Hydroxychloroquine, Azithromycin, Zinc, and Vitamin D3 With or Without Intravenous Vitamin C: An International, Multicenter, Randomized Trial

**DOI:** 10.7759/cureus.19902

**Published:** 2021-11-25

**Authors:** Karin Ried, Taufiq BinJemain, Avni Sali

**Affiliations:** 1 NIIM Research, National Institute of Integrative Medicine, Melbourne, AUS; 2 Health and Nutrition, Torrens University, Melbourne, AUS; 3 Discipline of General Practice, The University of Adelaide, Adelaide, AUS; 4 Gold Coast Clinic, National Institute of Integrative Medicine, Gold Coast, AUS; 5 NIIM Clinic, National Institute of Integrative Medicine, Melbourne, AUS

**Keywords:** vitamin d, intravenous vitamin c, zinc, hydroxychloroquine, covid-19 treatment, covid-19

## Abstract

Background

COVID-19 is a global pandemic. Treatment with hydroxychloroquine (HCQ), zinc, and azithromycin (AZM), also known as the Zelenko protocol, and treatment with intravenous (IV) vitamin C (IVC) have shown encouraging results in a large number of trials worldwide. In addition, vitamin D levels are an important indicator of the severity of symptoms in patients with COVID-19.

Objectives

Our multicenter, randomized, open-label study aimed to assess the effectiveness of HCQ, AZM, and zinc with or without IVC in hospitalized patients with COVID-19 in reducing symptom severity and duration and preventing death.

Methods

Hospitalized patients with COVID-19 in seven participating hospitals in Turkey were screened for eligibility and randomly allocated to receive either HCQ, AZM, and zinc (group 1) or HCQ, AZM, zinc plus IV vitamin C treatment (group 2) for 14 days. The patients also received nontherapeutic levels of vitamin D3.

The trial is registered on the Australian and New Zealand Clinical Trial Registry ACTRN12620000557932 and has been approved by the Australian Therapeutic Goods Administration (TGA).

Results

A total of 237 hospitalized patients with COVID-19 aged 22-99 years (mean: 63.3 ± 15.7 years) were enrolled in the study. Almost all patients were vitamin D deficient (97%), 55% were severely vitamin D deficient (<25 nmol/L) and 42% were vitamin D deficient (<50 nmol/L); 3% had insufficient vitamin D levels (<75 nmol/L), and none had optimal vitamin D levels.

Of the patients, 73% had comorbidities, including diabetes (35%), heart disease (36%), and lung disease (34%).

All but one patient (99.6%; n = 236/237) treated with HCQ, AZM, and zinc with or without high-dose IV vitamin C (IVC) fully recovered. Additional IVC therapy contributed significantly to a quicker recovery (15 days versus 45 days until discharge; p = 0.0069).

Side effects such as diarrhea, nausea, and vomiting, reported by 15%-27% of the patients, were mild to moderate and transient. No cardiac side effects were observed.

Low vitamin D levels were significantly correlated with a higher probability of admission to the intensive care unit (ICU) and longer hospital stay.

Sadly, one 70-year-old female patient with heart and lung disease died after 17 days in ICU and 22 days in the hospital. Her vitamin D level was 6 nmol/L on admission (i.e., severely deficient).

Conclusions

Our study suggests that the treatment protocol of HCQ, AZM, and zinc with or without vitamin C is safe and effective in the treatment of COVID-19, with high dose IV vitamin C leading to a significantly quicker recovery.

Importantly, our study confirms vitamin D deficiency to be a high-risk factor of severe COVID-19 disease and hospitalization, with 97% of our study’s patient cohort being vitamin D deficient, 55% of these being severely vitamin D deficient, and none had optimal levels.

Future trials are warranted to evaluate the treatment with a combination of high-dose vitamin D3 in addition to HCQ, AZM, and zinc and high-dose intravenous vitamin C.

## Introduction

The severe acute respiratory syndrome coronavirus 2 (SARS-CoV-2), or COVID-19, has affected millions of people worldwide. COVID-19 was first reported by the World Health Organization in December 2019 and was declared a worldwide pandemic in March 2020. Exploring therapies potentially of benefit for COVID-19 has been a public health emergency.

SARS-CoV-2 enters cells by binding to the ACE2 receptor. Higher blood levels of ACE2 reflect shedding from the myocardium and pulmonary epithelium and identify patients who are vulnerable to the development of life-threatening complications.

Early in the pandemic, the combination of hydroxychloroquine (HCQ), azithromycin (AZM), and zinc, also known as the Zelenko protocol, had shown great promise in the treatment of COVID-19 [1,2].

In vitro, chloroquine increases the endosomal pH required for the virus to fuse with cells and interferes with the glycosylation of SARS-CoV-2 cell receptors, thereby blocking viral infection [3,4]. Investigators performed a time-of-addition assay, which showed that chloroquine is effective at both the entry and post-entry stages of the SARS-CoV-2 infection in cells. Hydroxychloroquine has greater in vitro potency than chloroquine against SARS-CoV-2 and, because of its enhanced safety profile, can be given at higher doses than chloroquine [5].

As of October 2021, a meta-analysis of more than 290 worldwide trials involving more than 412,000 patients found that HCQ significantly reduced morbidity and mortality in patients with COVID-19. Specifically, when HCQ is used in early treatment, a meta-analysis of 32 studies involving more than 54,600 patients suggested HCQ to improve symptoms and prevent death by 64%-75% (all early treatment studies (n = 32): RR, 0.36 (0.29-0.46), p < 0.0001; early treatment studies reporting mortality (n = 13): RR, 0.25 (0.16-0.40), p < 0.0001) [6].

Azithromycin is a macrolide antibiotic that has been found to inhibit the viral tropism and replication of Zika and Ebola viruses [7,8]. An in vitro study has shown the activity of azithromycin (AZM) in combination with hydroxychloroquine (HCQ) against SARS-CoV-2 [9].

In addition, the effectiveness of this combination therapy of HCQ and AZM, when used early, as was demonstrated in a clinical study involving 83 patients in Turkey, reduced recovery time and shortened hospital length of stay [10].

In therapeutic doses, HCQ has a high safety profile and works as a zinc ionophore, enabling zinc to enter a virus-infected cell, increasing intracellular zinc concentrations [11].

Zinc itself has antiviral properties, boosting both innate and humoral immunity [12]. High intracellular concentrations of zinc are essential to inhibit viral replication and proliferation, including coronavirus RNA-dependent RNA polymerase activity [13].

The Zelenko COVID-19 treatment protocol consists of the following triple therapy for five consecutive days in addition to standard supportive care: zinc sulfate (220 mg capsule once daily, containing 50 mg elemental zinc), HCQ (200 mg twice daily), and AZM (500 mg once daily) [2].

In addition, intravenous vitamin C (IVC) has known immune-stimulating and antiviral properties [14] and had shown promise as a treatment for acute respiratory syndrome and pneumonia [15]. Recent studies reported on the benefits of IVC therapy for COVID-19 [16,17].

Furthermore, a large number of studies (n > 200) have demonstrated low vitamin D levels to be a risk factor for the severity of COVID-19 symptoms and hospitalization [18-20].

Adequate vitamin D levels are of great importance in the prevention of respiratory infections, as vitamin D protects against pathogens including viruses via the innate and adaptive immune systems, involving white blood cells and T-cells [21].

In our study, we aimed to assess the optimal treatment protocol for hospitals to consider in their treatment for patients with COVID-19, in order to reduce the severity and duration of symptoms and save lives. Patients presenting at hospitals with COVID-19 symptoms were randomly allocated to the Zelenko protocol (HCQ + AZM + zinc) or the Zelenko protocol plus IV vitamin C.

All enrolled patients also received supplementation of 5000 IU/day of vitamin D3, an adequate dose if levels of vitamin D are insufficient (51-75 nmol/L); however, this dose is considered inadequate for vitamin D deficiency (<50 nmol/L).

## Materials and methods

Trial design and participants

Our study is an international, multicenter, open-label, randomized controlled trial evaluating the efficacy and safety of therapies with hydroxychloroquine (HCQ), azithromycin (AZM), zinc, and vitamin D3 alone (group 1) or HCQ + AZM + zinc in combination with IV vitamin C (group 2) in hospitalized patients with COVID-19. For stage 1 of the trial, we aimed to recruit 200 patients.

The trial was conducted in Australia and Turkey between January and June 2021. Stage 1 of the trial took place primarily in Turkey and involved seven participating hospitals in Eskisehir, Elazig, Istanbul, Erzincan, and Izmir.

The trial was approved by the National Health and Medical Research Council (NHMRC)-endorsed National Institute of Integrative Medicine (NIIM) Human Research Ethics Committee in Australia, the Turkish Ethics Committees at the Ministry of Health in Turkey, and participating hospitals.

The trial is registered on the Australian and New Zealand Clinical Trial Registry ACTRN12620000557932 and has been approved by the Australian Therapeutic Goods Administration (TGA).

All eligible participants provided electronic written informed consent.

Inclusion criteria

The inclusion criteria were as follows: (1) age ≥ 18 years, (2) informed written consent, and (3) diagnosis of active symptomatic COVID-19 confirmed by polymerase chain reaction (PCR) testing via nasal and/or oral swab at the time of enrolment for quantitative PCR assessment.

Exclusion criteria

The exclusion criteria were as follows: (1) known G-6-PDH deficiency; (2) contraindication to hydroxychloroquine, azithromycin, or vitamin C, allergy to study interventions, epilepsy, serious hearing or visual problems, advanced liver disease, history of severe depression, calcium oxalate stones, and pregnant or lactating women; (3) already receiving hydroxychloroquine, azithromycin, vitamin C >3 g daily, or an experimental antiviral; (4) history of fever (e.g., night sweats and chills) and/or acute respiratory infection (e.g., cough, shortness of breath, and sore throat) of more than seven days’ duration; (5) calculated creatinine clearance of <30 mL/minute; (6) baseline electrocardiogram (ECG) showing QTc ≥ 470 for males and QTc ≥ 480 for females; and (7) receipt of a drug known to increase QTc, such as quetiapine, amiodarone, and sotalol.

Intervention

Group 1 received HCQ + zinc + AZM + vitamin D3, whereas group 2 received vitamin C + group 1 interventions. Hydroxychloroquine (HCQ) was given as 400 mg peroral (PO) once a day for one day, followed by 200 mg once a day for six days. Azithromycin (AZM) was given as 500 mg PO on day 1, followed by 250 mg PO once daily for four days. Zinc citrate was given as 30 mg elemental zinc PO daily for 14 days. Vitamin D3 was given as 5,000 IU PO daily for 14 days. IV vitamin C (sodium ascorbate) was given as 50 mg/kg every six hours on day 1, followed by 100 mg/kg every six hours (four times daily, 400 mg/kg/day) for seven days (average: 28 g/day; maximum dose: 50 g/24 hours for those weighing more than 125 kg).

Data collection

Project management and data collection were carried out by appointed teams at the participating sites.

The participants’ gender, age, disease severity, comorbidities (smoking, diabetes, heart disease, lung disease, and immunosuppression), other medications, and trial outcomes were entered into an electronic online database using Microsoft Forms questionnaires.

Outcomes

Primary Outcome

The primary outcome was mortality or need for invasive mechanical ventilation at any time in the first 15 days from enrolment.

Secondary Efficacy Outcomes

The secondary efficacy outcomes (measured at both 15 and 45 days from enrolment) are mortality, invasive mechanical ventilation, need for humidified high-flow oxygen, admission to the intensive care unit (ICU), days in the hospital, days in the ICU, renal replacement therapy, and extracorporeal support.

The secondary efficacy outcomes also include the World Health Organization (WHO) Master Protocol ordinal score at day 15 as follows: (1) not hospitalized, no limitations on activities; (2) not hospitalized, limitation on activities; (3) hospitalized, not requiring supplemental oxygen; (4) hospitalized, requiring supplemental oxygen; (5) hospitalized, on noninvasive ventilation or high-flow oxygen devices; (6) hospitalized, on invasive mechanical ventilation or ECMO; and (7) death.

Secondary Safety Outcomes

The secondary safety outcomes are QTc prolongation (>500 ms) 24 hours following the initial dose of study drugs, serious ventricular arrhythmia (including ventricular fibrillation) or sudden unexpected death in the hospital, and any of the following adverse events in the first 10 days from enrolment: diarrhea, grade 2 or greater; nausea, grade 2 or greater; and vomiting, grade 2 or greater (Appendices).

Adaptive design features

The study was overseen by the Steering Committee consisting of chief investigators (TB, KR, and AS) and investigators at recruited sites. Independent Data Safety Monitoring Committees (DSMC) at participating hospitals monitored the progress and safety of the trial treatment and were to make recommendations on whether to continue, modify, or stop the trial for safety or ethical reasons.

Sample size calculation

In stage 1, the sample size required is n = 100 in each intervention arm in order to have a statistical power of 80% to detect a relative risk reduction (RRR) of 30% in the proportion progressing to mechanical ventilation or death, compared with standard care, and assuming a standard-of-care risk of progression of 30%. Since the participants were hospitalized, we assumed minimal (<1%) loss to follow-up. The total sample size was n = 200.

Analyses were performed using IBM SPSS version 26. Statistical significance was set at p < 0.05. The primary analysis of efficacy was conducted under the intention-to-treat principle; all randomized participants were included in the analyses. Descriptive analysis was conducted on all variables. Any variable differences between groups were included in analyses as covariates. Differences between the groups and comparison of continuous outcome variables were analyzed using Student's t-test or analysis of covariance (ANCOVA) and chi-square analysis for dichotomous variables or Mann-Whitney U-tests for ranking variables. Correlations between variables were assessed using Pearson’s correlation coefficient.

## Results

**Figure 1:**
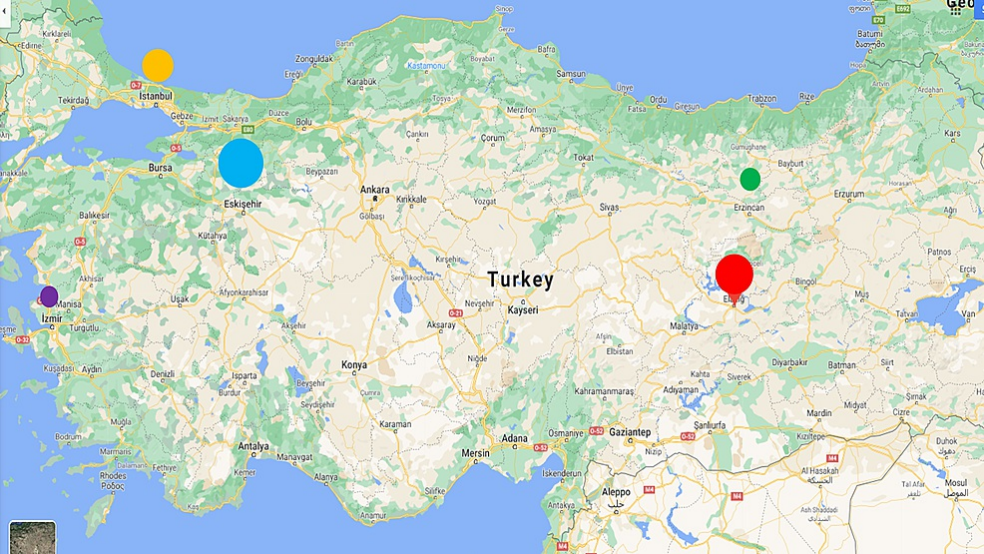
Participating hospital sites in Turkey

Seven hospital sites in Turkey participated in the multicenter trial (Figure 1).

Participants

In total, 237 hospitalized patients were enrolled in the study in Turkey. Table 1 outlines the number (%) of patients enrolled by hospital site.

**Table 1:** Number (%) of patients enrolled by hospital site

Hospital site	Color circle on the map (Figure 1)	Number of patients enrolled	% Patients
Eskisehir (ESOGU)	Blue	138	60%
Eskisehir (City Hospital)		26	
Elazig	Red	67	25%
Istanbul	Yellow	1	10%
Istanbul (University)		24	
Erzincan	Green	13	4%
Izmir	Purple	2	1%

The average age of the patients enrolled was 63.3 ± 15.7 years, ranging from 22 to 99 years. Half of the patients were male and half were female. All findings were independent of age and gender (Table 2).

Out of the enrolled patients, 96% tested positive by the COVID-19 PCR test; those who tested negative had severe respiratory symptoms, such as cough and difficulty breathing (9/10), or had tested positive with a COVID-19 antibody test (1/10).

A small number of patients had recently received a vaccination: 2/230 (3%) reported to have received a flu vaccination and 5/232 (2%) had received a COVID-19 vaccination at the time of the study.

Of the participants, 73% had comorbidities, including diabetes (35%), heart disease (36%), lung disease (34%) or were heavy smokers (41%), cancer (10%), or autoimmune disease (3%) (Table 2).

**Figure 2:**
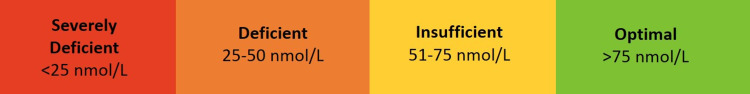
Vitamin D levels

Almost all hospitalized patients with COVID-19 enrolled in the study were vitamin D deficient (97%), 55% were severely vitamin D deficient (<25 nmol/L) and 42% were vitamin D deficient (<50 nmol/L); 3% had insufficient vitamin D levels (<75 nmol/L), and none had optimal vitamin D levels (Table 2, Figure 2).

**Table 2:** Participant characteristics M, male; f, female; N, number; SD, standard deviation; nmol/L, nanomole per liter; umol/L, micromole per liter; mg/dL, milligram per deciliter; BNP, brain natriuretic peptide; FDP, fibrinogen degradation product

Variable	N (%)/mean ± SD	Comment
Hospitalized patients/outpatients	237 (90%)/29 (10%)	Only hospitalized patients were included in the analysis.
Gender (M/F)	119 (50%)/118 (50%)	
Age (years)	63.3 ± 15.7 (range: 22–99) years	
COVID-PCR (positive/negative)	227 (96%)/10 (4%)	9/10 with negative PCR had severe respiratory symptoms (cough/difficulty breathing).
Flu/COVID-19 vaccination	7/230, 5/232	
Days unwell at enrolment	4 ± 3 (range: 1–30)	
Vitamin D level (nmol/L)	24.1 ± 9.2 (range: 2–64)	
Vitamin D categories (Figure 2)		
Severely deficient (<25 nmol/L)	131 (55%)	The majority were deficient in vitamin D; half were severely deficient.
Deficient (25–50 nmol/L)	99 (42%)	
Insufficient (51–75 nmol/L)	7 (3%)	
Optimal (>75 nmol/L)	None	
Zinc (umol/L)	17.6 ± 4.4 (range: 5–34)	
Deficient (<14 umol/L)	49 (21%)	
Normal (14–23 umol/L)	169 (71%)	
Excess (>23 umol/L)	19 (8%)	
Mg (mg/dL)	1.88 ± 0.27	
Deficient (<1.7 mg/dL)	48 (20%)	
Normal (1.7–2.2 mg/dL)	177 (75%)	
Excess (>2.2 mg/dL)	12 (5%)	

Variable	N (%)
Comorbidities (1+)	172 (73%)
Diabetes	82 (35%)
Heart disease	86 (36%)
Lung disease	56 (34%)
Heavy smoker	98 (41%)
Cancer	10 (4%)
Autoimmune disease	7 (3%)
Biomarkers (cardiopulmonary – abnormal)	
Prothrombin	28 (12%)
D-dimer	40 (17%)
Fibrin + FDP	37 (16%)
Troponin	46 (19%)
BNP	32 (14%)

Safety

During the study, the patients were regularly monitored with an electrocardiogram (ECG), and none reported any abnormalities or ventricular fibrillation.

A small proportion of the enrolled patients (12%-17%) had been admitted to the hospital with abnormal levels of troponin, D-dimer, fibrin, or brain natriuretic peptide (BNP), indicative of cardiopulmonary stress, likely due to acute infection with SARS-CoV-2 (Table 2). These biomarker levels either improved or did not worsen during the trial, and none of the trial participants suffered blood clots, stroke, or heart attack.

Adverse effects

Mild to moderate transient adverse events were reported by a proportion of the patients in both groups: diarrhea, 17%-27%; nausea, 18%-20%; and vomiting, 15%-16% (Table 3).

**Table 3:** Adverse effects HCQ, hydroxychloroquine; AZM, azithromycin; IVC, intravenous vitamin C

Symptom	Group 1 (HCQ + AZM + zinc); Group 2 (IVC + HCQ + AZM + zinc)	No symptoms (%)	Mild symptoms (%)	Moderate symptoms (%)
Diarrhea	1	72	25	3
	2	82	15	2
Nausea	1	80	20	0
	2	81	17	2
Vomiting	1	85	15	0
	2	84	15	1

Symptoms on admission

The enrolled patients experienced the following symptoms on admission into the study: 70% had difficulty breathing, of which 7% had severe difficulty breathing and 21% had moderate difficulties; 60% had headache (56% mild); 48% reported cough, of which 11% had a moderate cough and 3% had a severe cough; 46% had a fever, of which the majority were mild (40%); and 41% reported a loss of sense of smell.

Key findings

Almost all hospitalized patients with COVID-19 enrolled in the study were vitamin D deficient (97%), 55% were severely vitamin D deficient (<25 nmol/L) and 42% were vitamin D deficient (<50 nmol/L); 3% had insufficient vitamin D levels (<75 nmol/L), and none had optimal vitamin D levels (Table 2).

This finding is in line with the international literature, linking low vitamin D levels with higher susceptibility to symptomatic respiratory infection, including COVID-19.

In our trial, we found a statistically significant correlation between vitamin D levels and ICU admission. The lower the vitamin D level, the higher the probability of being admitted to the ICU (14.2 nmol/L (n = 13) versus 25.1 nmol/L (n = 224); p < 0.0001). Furthermore, we found a statistically significant correlation between lower baseline vitamin D levels and longer hospital stay (r = -0.195; p = 0.003). Vitamin D levels were comparable by gender and age.

Outcomes

All but one patient in our trial fully recovered, half (52%) of the participants after 15 days, and half (48%) at 45 days follow-up since enrolment (Table 4).

During the hospital stay, 40% of the patients required oxygen, and 6% were admitted to the ICU; one patient was treated on a ventilator, one required renal replacement, and one died (Table 4).

One 70-year-old female patient with heart and lung disease died after 17 days in the ICU and 22 days in the hospital; her vitamin D level was 6 nmol/L on admission (<25 nmol/L = severely deficient).

**Table 4:** Outcomes

Variable	N (%)	Comment
During hospital stay		
Requiring supplemental oxygen	82 (40%)	
ICU admission	13 (6%)	
Ventilator	1	
Renal replacement	1	
Died	1	Vitamin D = 6 nmol/L
Day 15 follow-up		
Total recovery/no/mild symptoms	120 (52%)/45 (19%)/68 (29%)	
Day 45 follow-up		
Total recovery	236 (99.6%)	

A larger proportion of participants in group 2, which received additional IV vitamin C therapy, were symptom-free and discharged from the hospital earlier on day 15 compared with day 45. This difference was statistically significant, indicating that IV vitamin C treatment contributes significantly to quicker recovery (day 15 versus day 45; p = 0.0069) (Table 5).

**Table 5:** Outcomes by treatment group HCQ, hydroxychloroquine; AZM, azithromycin; IVC, intravenous vitamin C Chi-square statistical analysis: IV vitamin C treatment contributed significantly to quicker recovery (day 15 versus day 45; p = 0.0069).

		Total recovery (symptom-free, discharged from the hospital); N (%)	
Group	All N (%)	Day 15	Day 45
1 (HCQ + AZM + zinc)	75 (32%)	29 (39%)	46 (61%)
2 (IVC + HCQ + AZM + zinc)	162 (68%)	93 (57%)	68 (42%)
Total	237	122 (51%)	114 (49%)

## Discussion

Our study suggests that the combination of hydroxychloroquine (HCQ), azithromycin (AZM), and zinc with or without IV vitamin C is safe and effective in the early treatment of COVID-19. No cardiac side effects were observed.

All but one patient (99.6%; n = 236/237) in our trial fully recovered, with IV vitamin C contributing to a significantly quicker recovery (15 days versus 45 days until discharge).

Our study’s findings are in line with the international literature, whereby the treatment of COVID-19 with HCQ, zinc, and AZM or intravenous vitamin C has shown to be effective in aiding recovery and reducing mortality.

The effectiveness of HCQ with or without AZM in the treatment of COVID-19 has been demonstrated in a meta-analysis of more than 290 trials involving more than 412,000 patients, whereby improvement of symptoms and prevention of death were achieved at 64%-75% if treatment was provided early [6].

The combination of HCQ, AZM, and zinc, also known as the Zelenko protocol, has been shown to reduce hospitalization and mortality significantly, whereby a significantly smaller number of patients in the treatment group was hospitalized or died compared with the untreated group (hospitalized: 3% treated versus 15% untreated; died: 0.7% treated versus 3.4% untreated) [2].

To date, few studies have looked into the effectiveness of early treatment of patients with COVID-19 with vitamin C/ascorbic acid [22]. One study found that high-dose intravenous vitamin C provided a 72% improvement in symptoms and reduced recovery time [17]. In contrast, a study that used an oral combination of vitamin C and zinc did not find a significant difference in the improvement of symptoms between the treatment and the control group [23].

Our study is the first to combine HCQ, AZM, and zinc with high-dose intravenous vitamin C therapy, resulting in the total recovery of 99.6% of participants, whereby IVC contributed to a significantly quicker recovery and discharge from the hospital. The treatment protocol was highly tolerable and did not cause any cardiac complications.

Importantly, our study confirmed vitamin D deficiency to be a high-risk factor of severe COVID-19 disease and hospitalization, with 97% of our study’s patient cohort being vitamin D deficient, of which 55% were severely vitamin D deficient.

This finding is in line with the international literature, highlighting the importance of adequate vitamin D levels for immune function, prevention of acute respiratory infections including COVID-19, and recovery [18,19,24,25].

Specifically, vitamin D protects against pathogens including viruses via the innate and adaptive immune system, involving white blood cells and T-cells [21].

Several studies conducted earlier in the pandemic have linked vitamin D deficiency with the risk and severity of COVID-19 infection and hospitalization [26,27], while a recent systematic review and meta-analysis of eight studies and >1500 participants concluded that vitamin D levels over 50 nmol/L can reduce the mortality risk of COVID-19 to zero [28].

It is known that a large proportion of Australians are vitamin D deficient [29]. Research has proven vitamin D supplementation to be a key factor to alleviate vitamin D deficiency and subsequently to prevent the onset and severity of acute respiratory tract infections and reduce morbidity and mortality [20].

Higher daily doses of 5000-10000 IU vitamin D3 orally are considered safe and effective in elevating vitamin D deficiency [19].

Furthermore, our study revealed that lower vitamin D levels were significantly correlated with a higher probability of being admitted to the ICU, leading to a significantly longer hospital stay.

While comorbidities contribute to the risk of hospitalization [30] (three-quarters of our study population had comorbidities), severe vitamin D deficiency (6 nmol/L) was the most probable cause for the death of the 70-year-old patient with lung and heart disease.

## Conclusions

In summary, our study found vitamin D deficiency to be a high-risk factor for severe COVID-19 disease and hospitalization, with 97% of our study’s patient cohort being vitamin D deficient, of which 55% were severely vitamin D deficient, and none had optimal levels. In addition, vitamin D levels were significantly correlated to ICU admission and longer hospital stay.

Furthermore, our study contributes to the evidence of HCQ, AZM, and zinc with or without IV vitamin C being safe and effective in the treatment of COVID-19, with IV vitamin C contributing to a significantly quicker recovery.

Future research based on the findings in stage 1 of our trial in line with the international literature of the importance of adequate vitamin D levels to immune function and recovery are encouraged to adapt the protocol for the next stage of the trial by adding a high-dose vitamin D3 to all enrolled patients.

## Appendices

Definitions of Adverse Events

Diarrhea

Grade 1: Increase of <4 stools per day over baseline; mild increase in ostomy output compared with baseline

Grade 2: Increase of 4-6 stools per day over baseline; moderate increase in ostomy output compared with baseline; limiting instrumental ADL

Grade 3: Increase of ≥7 stools per day over baseline; hospitalization indicated; severe increase in ostomy output compared with baseline; limiting self-care

Grade 4: Life-threatening consequences; urgent intervention indicated

Nausea

Grade 1: Loss of appetite without alteration in eating habits

Grade 2: Oral intake decreased without significant weight loss, dehydration, or malnutrition

Grade 3: Inadequate oral caloric or fluid intake; tube feeding, TPN, or hospitalization indicated

Vomiting

Grade 1: Intervention not indicated

Grade 2: Medical intervention indicated

Grade 3: Tube feeding, TPN, or hospitalization indicated

Grade 4: Life-threatening consequences
